# Autohydrolysis of *Lentinus edodes* for Obtaining Extracts with Antiradical Properties

**DOI:** 10.3390/foods9010074

**Published:** 2020-01-09

**Authors:** Liceth Rocío Huamán-Leandro, María Jesús González-Muñoz, Catalina Fernández-de-Ana, Arturo Rodríguez-Blanco, María Dolores Torres, Herminia Domínguez

**Affiliations:** 1 Departamento de Enxeñería Química, Universidade de Vigo (Campus Ourense), Edificio Politécnico, As Lagoas, 32004 Ourense, Spain; rocioliz15@hotmail.com (L.R.H.-L.); mjgm@uvigo.es (M.J.G.-M.); herminia@uvigo.es (H.D.); 2 CITI-Universidade de Vigo, Tecnopole, San Cibrao das Viñas, 32901 Ourense, Spain; 3 Hifas da Terra SL, Portamuiños,7, 36154 Bora Pontevedra, Spain; investigacio@hifasdaterra.com (C.F.-d.-A.); investigacion@hifasdaterra.com (A.R.-B.)

**Keywords:** *Lentinus edodes*, autohydrolysis, hydrogels, saccharide components, radical scavenging

## Abstract

The autohydrolysis of *Lentinus edodes* was proposed for the extraction of components with antioxidant properties. Operation under non-isothermal conditions was evaluated and compared with isothermal heating. The influence of process severity was assessed in the range of 0.18 to 4.89 (temperature between 50 and 250 °C), up to 80% (d.b.) The influence of process severity during the autohydrolysis of *Lentinus edodes* was assessed in the range −0.3 to 4.89 (temperature between 50 and 250 °C). Up to 80% (d.b.) of the initial raw material could be solubilized at 210 °C. The different behavior of the saccharide and phenolic fractions was observed with the treatment temperature. Whereas the highest concentration of the saccharide components (mainly glucooligosaccharides) was found at 210 °C, the maximum phenolic yield was identified at 250 °C. The phenolic content and the antiradical properties of the extract showed a continuous increase with the temperature range studied, and at 250 °C, showed antiradical properties comparable to synthetic antioxidants. Autohydrolysis liquid fractions were used as solvents in the formulation of bioactive starch-based hydrogels, identifying a positive correlation between the gel softening and the extracts’ bioactivity features.

## 1. Introduction

The basidiomycetous *Lentinula edodes* (shiitake) is the second most popular worldwide cultivated edible mushroom. Shiitake has high nutritional value and is one of the most important sources of β-glucans, with immunomodulatory and anti-tumor properties [1,2,3]. Mushroom polysaccharides are the structural components of the cell wall, composed of two main polymers, chitin and β-glucans, and smaller amounts of some other saccharides, i.e., trehalose and mannan oligosaccharides [4]. Molecules of β-1,3-glucan are relatively resistant to the acid in the stomach, pass slowly into the duodenum, and are entrapped by macrophage receptors. Lentinan enhances the resistance of intestinal mucosa to inflammation and inhibits the development of intestinal ulcers [4], but further research is required to know the mechanisms of the in vivo action of glucans.

Despite the popularity and the positive effects of fungi, the risk associated with their consumption, mainly derived from the presence of noxious substances such as heavy metals, has been emphasized. To overcome this problem, the selective extraction and purification of the active fractions is proposed. Some cases of erythema arisen after shiitake intake are thought to be due to lentinan, but good prognosis and complete recovery were reported [5].

Several kinds of polysaccharides have been identified in *Lentinula edodes* with different extraction and purification processes [2,3]. The β-glucan content in *L. edodes* is lower than in some *Pleurotus* sp., but the water-soluble fraction is much higher. In addition, the water-soluble form has a more pronounced effect on the immune systems of humans and animals [4], and soluble polar substances present in *L. edodes* extracts show antioxidant and anticancer properties [6].

Classic extraction procedures of D-glucan from *L. edodes* use acid and hot water [7]. The hot water (95 °C) extracts from the 95% ethanol extracted fruiting bodies, once purified, show potent antioxidant activity in a dose-dependent manner [8].

Accelerated solvent extraction (ASE) at 10.7 MPa and 25 °C in 15 cycles of 5 min was efficient to obtain aqueous fractions with high 3-hydroxy-3-methyl-glutaryl CoA reductase (HMGCR) inhibitory activity from *L. edodes* [9]. A pressurized water extraction method at 200 °C, five cycles of 5 min each at 10.3 MPa, was used to extract glucans from *L. edodes* with hypocholesterolemic properties and bile acids-binding capacities similar to a β-glucan enriched fraction obtained from cereals [10].

Pressurized water extraction, at 2.5–25.3 MPa and 28 °C, was reported for polysaccharides from *L. edodes* to improve the extraction efficiency and to retain the general structures and bioactivity of the polysaccharides [1]. The water extraction under pressurized conditions can cause partial destruction of the hydrogen bonding of solid crude polysaccharides; the structure elasticity increased the water solubility of polysaccharides [1] and thermal softening [11]. Thermal extraction of glucans using hot compressed water occur via hydrolysis, dehydration, condensation and dehydrogenation, and deoxygenation [11].

Subcritical water (SW) extraction or water under pressurized conditions is regarded as a “green” processing method and has received increased attention as an alternative to conventional technologies. SW can be used to extract polar organic compounds or to aid in the hydrolytic fractionation of lignocellulosic materials. In this catalytic process, known as autohydrolysis, the acids from the hydrolysis of acetyl and uronic groups cause the solubilization of carbohydrates. Hot compressed water at temperatures of 100–190 °C and a pressure of 4.0 MPa was utilized for the extraction of polysaccharides from *Ganoderma lucidum* [11], subcritical water at 121–150 °C increased the antioxidant activity of extracts from *Grifola frondosa* [12], at 50–300 °C was effective for *Inonotus obliquus* [13], and at 200–300 °C for *Pleurotus citrinopileatus* [14]. Hot compressed water treatment is particularly suitable for high-moisture biomasses since energy can be saved because drying is not necessary [11].

Autohydrolysis has not been tried with Shiitake and the present study aims at evaluating the effect of the final temperature during a hydrothermal treatment of *L. edodes* on the solubilization yield, chemical composition, and antiradical properties of the extracts. In parallel, the potential of *L. edodes* autohydrolysis liquors as a solvent in the development of functional starch-based hydrogels for food and non-food applications are explored based on their mechanical rheological properties.

## 2. Materials and Methods

### 2.1. Raw Material

Shiitake mushrooms (*Lentinula edodes*) from a local shop (Ourense, Spain) were pre-dried in an oven at 50 °C and then forced-air-dried at 60 °C. Subsequently, the dehydrated mushrooms were milled to powder, homogenized, and stored in a dried place at room temperature and darkness.

### 2.2. Chemical Characterization

Samples from the homogenized mushroom lot were subjected to moisture determination in an oven at 105 °C during 24 h (method ISO 638), to ash determination in a furnace at 525 °C during 6 h (method ISO 776), to extractives determination in a Soxhlet unit with ethanol. Fatty acids were determined by GC-MS (QP 2010, Shimadzu, Japan) using a SP-2380 capillary column (Supelco, 30 m × 0.32 mm, ID 0.25 µm film thickness), with 1.2 mL He/min, an injector temperature of 250 °C, and a temperature ramp of 4 °C/min from 50 to 250 °C and keeping at 250 °C for 15 min. The injection was 1 µL with a split ratio of 100, after Soxhlet extraction with hexane and derivatization by methylation (method ISO 12966-3), and to total nitrogen determination by elemental analysis in ThermoFinnigan Flash EA 1112 series equipment. Quantitative acid hydrolysis in two steps, with sulphuric acid at 72% in the first stage and 4% in the second stage (method TAPPI T13m), was carried out in order to determine carbohydrates by HPLC using the same method as for the authohydrolysis liquors (see Section 2.4). The nitrogen and the fatty acids determinations were carried out in the “Centro de Apoyo Científico y Tecnológico a la Investigación” (CACTI) of the University of Vigo.

### 2.3. Autohydrolysis

Mixtures of mushroom powder with water at a liquid-to-solid mass ratio of 12:1 was subjected to temperatures from 50 to 250 °C, at 160 rpm speed agitation, in a 600 mL pressurized reactor (Series 4560, Parr Instrument Company, Moline, IL, USA). All experiments were carried out under non-isothermal conditions; once the maximum temperature was reached, the system was cooled, except for the experiment at a maximum temperature of 50 °C, which was kept isotherm for 7.5 min. Once the processes were finalized, the system was allowed to cool and the solid and liquid phases were separated by filtration.

### 2.4. Analysis of Liquors

Aliquots of the liquors at each temperature were filtered through 0.45 µm cellulose acetate membranes and analyzed by High Performance Liquid Chromatography – infrared spectroscopy (HPLC-IR) using two different columns, which achieves a satisfactory resolution of peaks which are overlapped in one or other column. Samples were also subjected to post-hydrolysis with 4% H_2_SO_4_ to hydrolyze the oligomers and to evaluate the content of oligomers in liquors by the difference between the content of monomers in the autohydrolysis liquor and the posthydrolysis liquor.

An Aminex HPX-87H column (Biorad, Richmond, VA, USA), with 0.003 M H_2_SO_4_ at 0.6 mL/min as the mobile phase operating at 50 °C was used to determine glucose, mannitol, glucuronic acid, and trehalose, and a CarboSep CHO 682 column (Transgenomics) with ultra-pure water at 0.4 mL/min as a mobile phase at 80 °C was used to determine glucose, xylose, galactose, mannose, fucose, and trehalose. Arabitol overlapped both with fucose in the Aminex HPX-87H column and with mannitol in the CarboSep CHO 682 column, so it was quantified by difference. Samples analyzed in the CarboSep column were previously neutralized with BaCO_3_.

The total phenolic content of liquors was determined by the Folin–Ciocalteau method [15].

Two methods based on radical scavenging were selected for measuring the antioxidant capacity of liquors: the 2,2-diphenyl-1-picrilhidrazilo DPPH assay [16] and the radical scavenging activity (ABTS)/ Trolox equivalent antioxidant capacity (TEAC) assay [17].

### 2.5. Severity Factor

In order to evaluate the effect of the operational conditions and to minimize the effect that a different heating-cooling profile could have in the results, a severity factor was used. This parameter, originally known as reaction ordinate, Ro, was introduced by Overend and Chornet [18] for modeling empirically the depolymerization of lignocellulosic. Abatzoglou, Chornet, and Belcacemi [19] obtained the same equation for explaining complex reaction systems based on a mechanistic approach considering a set of first-order kinetic equations and Arrhenius temperature dependence. Ro includes the effects of both time and temperature and provides an easy way to compare results among experiments carried out under different operational conditions.

Ro can be calculated using Equation (1):

(1) $$R_{o}\;=\;\int_{0}^{t}\exp\left(\frac{T-T_{r}}{\omega }\right)dt$$

where *T* is temperature (K), *t* is time (min), *Tr* is a reference temperature (373 K), and *ω* is a parameter related to the activation energy, with a commonly established value of 14.75 K. 

Usually, the severity, *S*, is expressed as the decimal logarithm of *Ro*:

(2) $$\;S\;=\log(R_{o})$$

### 2.6. Development of Functional Hydrogels

Potato starch extracted from local waste sources featuring low sizes was selected as a gelling agent, following the procedure previously reported [20]. The hydrogels were formulated following the procedure detailed elsewhere [21] at a biopolymer content (10 g/L) commonly used in food applications [22]. Briefly, the starch was dispersed in 100 mL of *Lentinus edodes* liquid extracts collected by autohydrolysis and stirred for 10 min at room temperature until homogeneity of the system. Samples were heated up to 70 °C and kept for 10 min to favor suitable sample gelatinization. Note here that prepared hydrogels were cold stored in a fridge at 4 °C for 24 h to ensure full hydrogel development. It is worth noting that the rheological features were determined after 1 h equilibration at room temperature.

### 2.7. Rheological Testing

Small amplitude oscillatory shear testing (SAOS) of the above hydrogels was conducted at least in triplicate on a controlled stress rheometer (MCR302, Anton Paar, Germany). The selected measuring system was a sandblasted plate–plate with a diameter of 25 mm. Formulated hydrogels were loaded on the measuring systems (1 mm of gap) and the edges of the samples sealed with paraffin oil. Before rheological testing, hydrogels were rested 5 min to favor thermal equilibration. Initially, stress sweeps were accomplished as reported elsewhere [23] for other starchy materials to define the linear viscoelastic region (below 30 Pa). Monitoring of elastic (G′) and viscous (G″) moduli with frequency was performed at 25 °C within the linear viscoelastic region (at 15 Pa).

### 2.8. Statistical Analysis

All measurements were made at least in triplicate. One-way analysis of variance was carried out by means of the PASW Statistics software (IBM SPSS Statistics 22.0, (IBM, Armonk, New York, United States)). Whenever the statistical analysis led to differences between the average values, a post-hoc Scheffé test was required to distinguish means with a confidence of 95% (*p* ≤ 0.05).

## 3. Results and Discussion

### 3.1. Chemical Characterization

The composition of *Lentinula edodes* is shown in Table 1. Carbohydrates account for 54.1% *w*/*w* of the dry matter (25.9% of these carbohydrates are polyols (mannitol and arabitol), and 52.7% are glucans). Other components are present in lower amounts: 24.3% *w*/*w* protein, 2.48% *w*/*w* fatty acids (77.7% linoleic acid and 22.3% palmitic acid), 1.44% *w*/*w* acid-insoluble matter, 8.13% *w*/*w* ashes, and 9.50% *w*/*w* are other non-determined compounds. The ethanolic extract of the ground dry mushroom was 19.2% *w*/*w*.

### 3.2. Autohydrolysis

#### 3.2.1. Severity Factor

**Figure 1:**
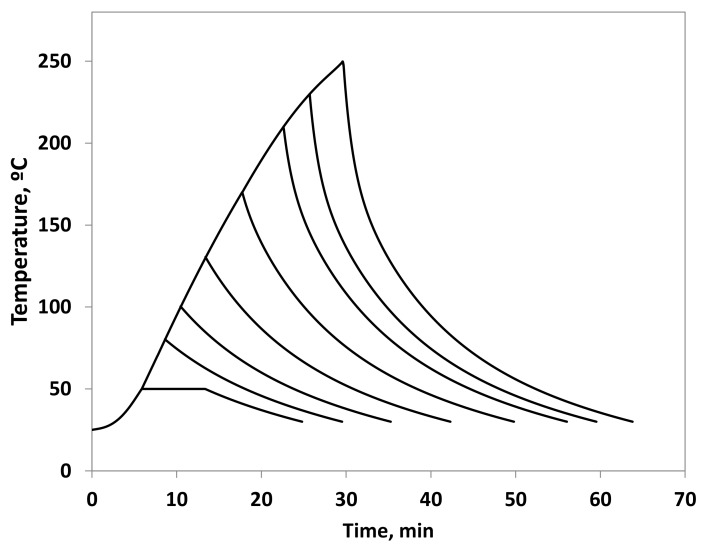
Temperature profile of each experiment.

The temperature profiles of each experiment to calculate the severity factor are represented in Figure 1, and the corresponding Ro and severity, S, are shown in Table 2. The Ro values were calculated considering initial and final temperatures of 30 °C, although the contribution of low temperatures on the Ro value is very low.

#### 3.2.2. Solubility Yield

It has been reported that the cell wall structure of the *L. edodes* fruit body is composed of the outside layer (an heteropolysaccharide with β-(1→3)-glucan and β-(1→6) branches, which can be extracted by water and diluted alkalis), the middle layer (mainly β-(1→6)-glucan with a small number of β-(1→3) branches, which is water-insoluble and can be extracted by strong alkalis), and the inner layer (a complex of chitin, β-glucan, and a small amount of acid polymer) [24]. The chemical structures of polysaccharides can vary with extraction technologies, and a successive breaking from the outer layer to the inner layer with mild-to strong extraction conditions has been suggested [3]. These authors showed higher peak intensities of water-soluble products from *G. lucidum* at 190 °C than those obtained at 100 and 140 °C, corresponding to hemicellulose groups but monomer units were not extracted by hot compressed water. A gradual increase in the concentration of carbon with increasing temperature was also noticed [11]. Optimal conditions for pressurized water extraction of β-glucans, α-glucans, and other hetero-/proteo-glucans from *Agaricus bisporus*, *Lentinula edodes*, and *Pleurotus ostreatus* were found at 10.3 MPa, 200 °C in 5 cycles of 5 min [10].

**Figure 2:**
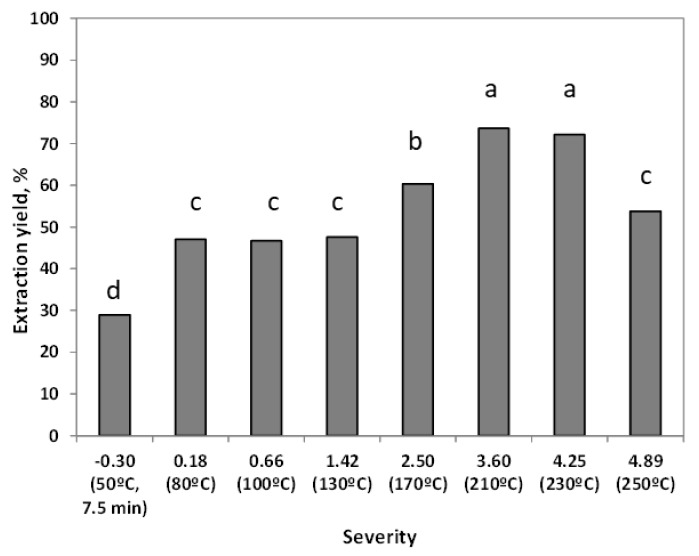
Effect of severity on the solid extraction yield. Error bars were lower than the symbol size. Letters represents the statistical analysis.

The effect of the severity (or maximum autohydrolysis temperature in the range 50–250 °C) on the treatment of ground *L. edodes* is shown in Figure 2. A steady increase in the solubilization yield was observed up to 210 °C with a maximum of 73.6%; however, lower values were observed at harsher treatments, since part of the solubilized compounds are decomposed to volatile compounds that could not be measured.

#### 3.2.3. Saccharide Composition

**Figure 3:**
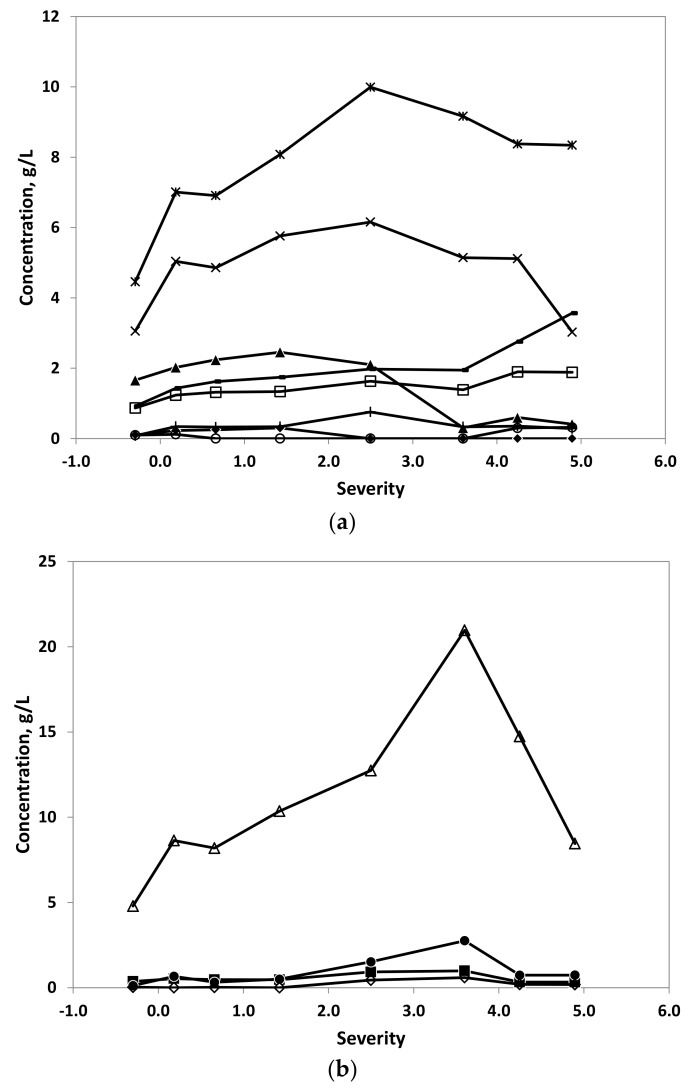
Effect of severity on the saccharide composition of autohydrolysis liquors. (**a**) monosaccharides: (▲) glucose, (♦) xylose, (○) mannose, (+) fucose; disaccharide: (x) trehalose; sugar derivatives: (□) glucuronic acid, (*) mannitol and (-) arabitol; (**b**) oligosaccharides: (∆) glucooligosaccharides (including trehalose), (◊) xylooligosaccharides, (●) mannoligosaccharides, (■) galactooligosaccharides. Error bars were lower than the symbol size.

The influence of the autohydrolysis severity (temperature–time) on the concentration of saccharide components (including monosaccharides, disaccharides, oligosaccharides, and sugar derivatives) in autohydrolysis liquors is shown in Figure 3a,b.

The content of carbohydrates in autohydrolysis liquors increases linearly from the milder conditions to the severity of 3.6 (T_max_ = 210 °C) varying from 34.4 to 47.3 g/100 g extract. At higher severity values, the content of carbohydrates decreases due to the degradation of saccharide.

The main saccharide components in autohydrolysis liquors are oligosaccharides made up of glucose, which increase continuously up a maximum value of 21.0 g/L (36.3 g galactooligosaccharides (GOS)/100 g of extract) at severity of 3.6 (T_max_ = 210 °C). At higher severity, the concentration decreases abruptly. The content in GOS includes the dimer trehalose (an alpha-linked disaccharide formed by an α,α-1,1-glucoside bond between two α-glucose units), with concentrations ranging from 4.9 to 6.2 g/L between 70 and 230 °C (S = 0.18 to 4.25) with the maximum at T_max_ = 180 °C (S = 2.5). At the softer and harsher conditions studied, the value of trehalose decreases to 3.0 g/L.

Mannitol is the second component in abundance in autohydrolysis liquors, with a maximum of 10.0 g/L at S = 2.50 (T_max_ = 180 °C), decreasing slowing at harsher conditions up to a value of 8.3 g/L. On the contrary, the content of arabitol increases slightly from 50 to 210 °C, and the increase is sharper from 210 to 250 °C. The concentrations vary from 0.9 to 3.6 g/L.

Regarding monosaccharides, the most abundant is glucose, with maximum of 2.5 g/L reached at Tmax = 130 °C (S = 1.42). From 180 °C (S = 2.5), the degradation of glucose is faster, obtaining concentrations lower than 0.6 g/L at severities higher than 3.6.

The rest of monosaccharides are less abundant, with concentrations lower than 0.76 g/L in all cases. The sugar acid glucuronic acid showed concentrations ranging from 0.9 to 1.9 g/L, increasing linearly from soft to harsh conditions.

The concentration of other sugars forming part of oligosaccharides is low. There is a slight increase from severities from 1.4 to 3.6 (T_max_ = 130–210 °C), decreasing at more severe conditions. The maximum concentration of manooligosaccharides was 2.76 g/L, 0.99 g/L for xylooligosaccharides, and 0.59 g/L for galactooligosaccharides.

#### 3.2.4. Total Phenolic Content and Antioxidant Properties of Autohydrolysis Liquors

**Figure 4:**
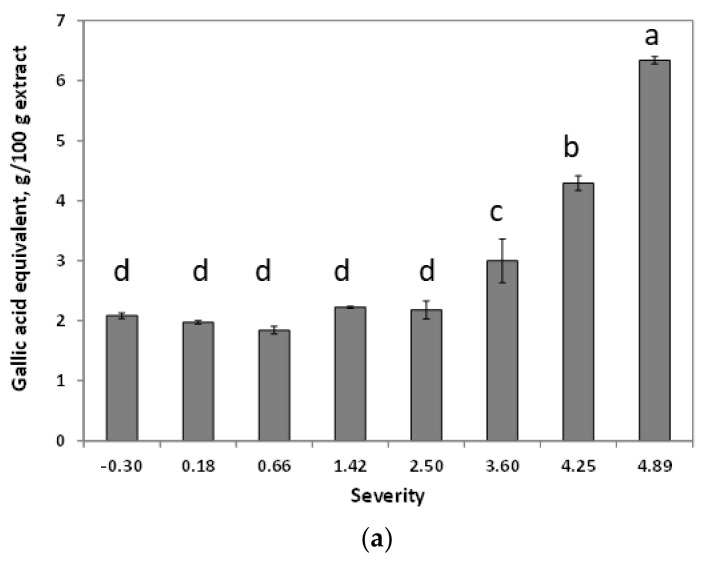

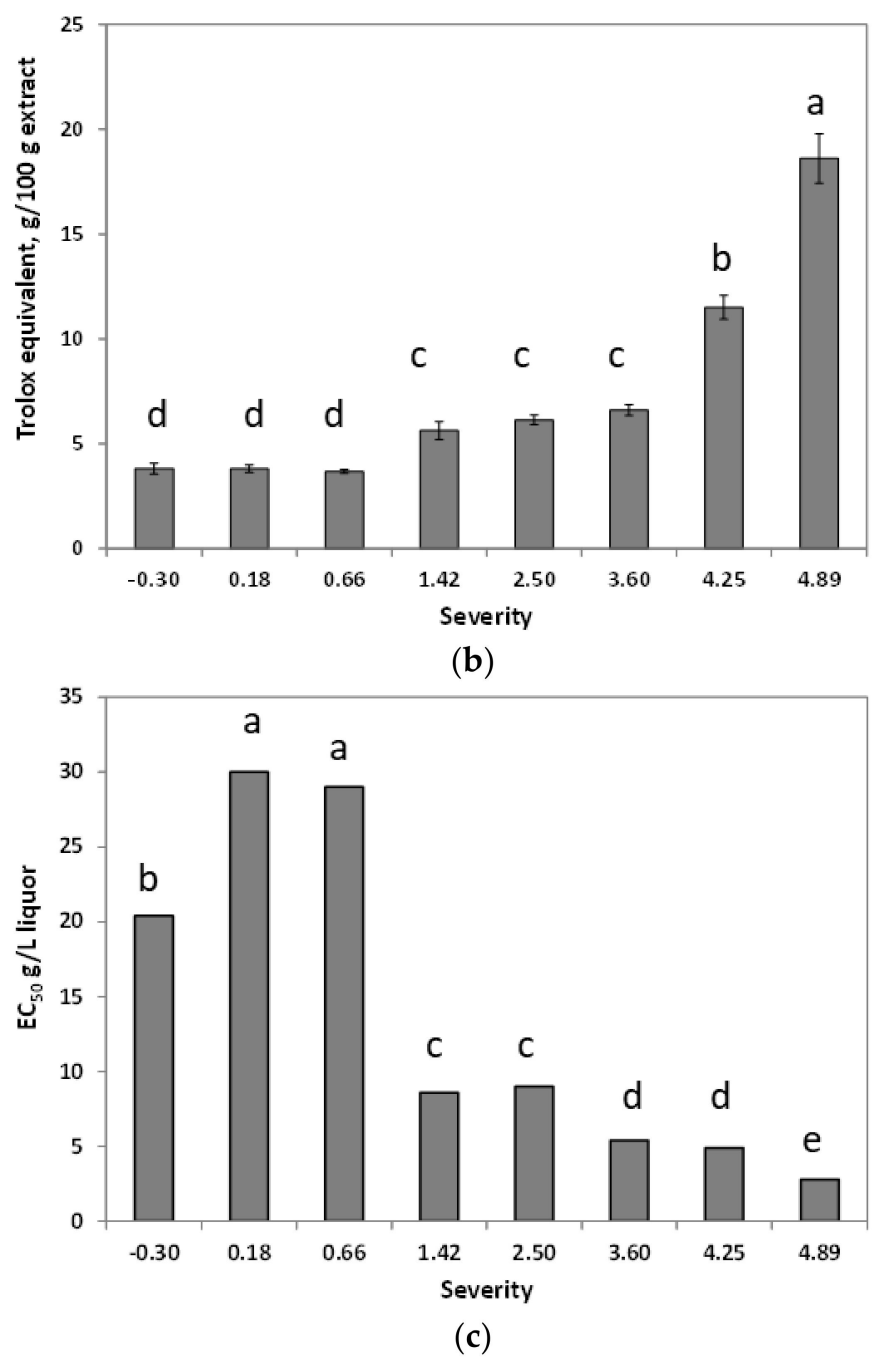
Effect of severity on (**a**) phenolic content of autohydrolysis liquors; (**b**) ABTS^+·^ radical scavenging capacity of autohydrolysis liquors, expressed as Trolox equivalents (TEAC); (**c**) DPPH^·^ radical scavenging capacity of autohydrolysis liquors, expressed as EC_50_. Letters represents the statistical analysis.

The total phenolic content (TPC) of the extracts (Figure 4a) showed a continuous increase at temperatures higher than 100 °C (severity > 0.66), which resulted in higher radical scavenging properties against ABTS ((2,2′-azino-bis(3-ethylbenzothiazoline-6-sulfonic acid)) and DPPH (2,2-diphenyl-1-picryl-hydrazyl-hydrate) (Figure 4b,c). Phenolic compounds accounted for 6.3 g gallic acid equivalents (GAE)/100 g extract (3.4 g GAE/100 g dry raw material) at the maximum severity tested (corresponding to T_max_ = 250 °C) and at this severity the antioxidant capacity was 18.6 g Trolox eq/100 g extract (10.0 g Trolox eq/100 g dry raw material).

These phenolic extraction yields are higher than those reported during the hydrothermal extraction of *L. edodes* at 100 °C for 30 min (37.5 mg/100 g raw material) and at 121 °C for 30 min (54.6 mg/100 g raw material (wet weight basis)) [25] and *Grifola frondosa* at 150 °C (13.61 mg GAE/g extract) [12]. Also Seo et al. [13] reported 18 time more efficient solubilization of *I. obliquus* at 250 °C for 30 min than at 50 °C for 10 min.

The EC_50_ for DPPH^·^ radical scavenging decreases with severity (see Figure 4c); the higher the severity, the higher the antiradical properties of the extract, following the same trend than with the ABTS^+·^ radical. The EC_50_ value of the extracts produced at 250 °C was comparable to the value for BHT (butylhydroxytoluene).

Higher temperatures were not tested in the present work, but probably the increase in the antiradical properties would not be indefinite, as reported during the subcritical water extraction of *Inonotus obliquus*, in the range 50–300 °C for periods up to 60 min in reaction vessels of stainless steel, using a mushroom:water ratio of 0.2 g:100 mL. The total phenolic content, reducing power, and DPPH^·^ and ABTS^+·^ radical scavenging activities increased with temperature and time, up to a maximum at 250 °C for 30 min but the activities decreased at 300 °C [13]. The TPC was maximum in the extracts obtained at 250 °C for 30 min, and the value was 18 times higher than that of the extract obtained at 50 °C for 10 min. A different behavior among fractions has been reported, i.e., optimal temperature differed for ABTS radical scavenging activity (300 °C) and for β-glucan content (200 °C) during subcritical water extraction of *Pleurotus citrinopileatus* [14], and for maximum phenolic extraction (150 °C) and β-glucan content during hydrothermal extraction of *Grifola frondosa* [12].

The reducing and radical scavenging properties of the supercritical water extracts from *I. obliquus* also increased as extraction temperature and times increased, the maximum at 250 °C for 60 min, with values comparable or higher than for L-ascorbic acid [13].

Heat treatment converted insoluble to soluble phenolic compounds by cleavage of the covalently bound phenolic compounds, and the increased content of free phenolic compounds was probably due to (i) changes in their extractability due to the disruption of the cell wall and liberation of antioxidant compounds from the insoluble portion of mushroom [13], (ii) the formation of novel compounds with antioxidant properties, i.e., non-enzymatic browning reaction products, and (iii) deactivation of endogenous oxidative enzymes [25]. The beneficial effect of thermal treatments was also reported, since increased levels of overall antioxidant activity and polyphenolic compounds of Shiitake extract were found with increasing heating temperature in the range 100–121 °C and from 15 to 30 min in an autoclave [25]. Even hot air drying at 50 °C resulted in high total phenolic, amino acid, uronic acid and neutral sugar contents, and antioxidant activities compared to freeze-drying and shade drying [26]. 

The phenolic components from *L. edodes* are responsible for hepatoprotective activity without cytotoxicity and with few side effects [27], and water-soluble polysaccharide crude and fractions [8], but in addition, several bioactive compounds including triterpenoids, sterols, vitamins and minerals had been isolated and identified from the fruiting bodies, mycelia, and culture medium of this mushroom.

The DPPH radical scavenging activities of the free extracts increased with the thermal treatment but the DPPH· scavenging activity of bound compounds was decreased and the ABTS^+·^ scavenging activity was doubled with respect to that of the raw Shiitake samples [25].

### 3.3. Rheological Behaviour of Developed Hydrogels

**Figure 5:**
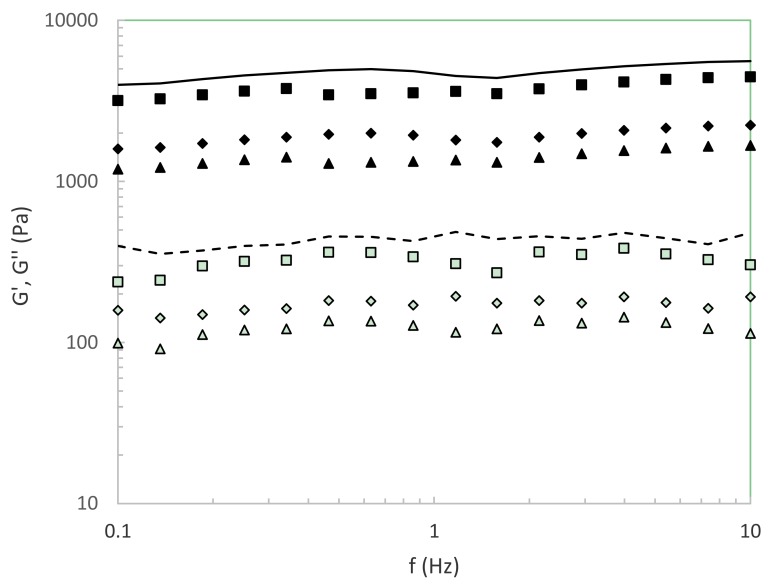
Viscoelastic features of functional starch-based hydrogels (10% *w*/*w*) made with liquors at representative autohydrolysis temperatures (°C): 50 (■), 210 (▲), and 250 (♦). Symbols: Elastic modulus (G′) (closed) and viscous modulus (G″) (open), lines represent the viscoelastic properties of starch-based hydrogels prepared in distilled water.

Figure 5 shows the viscoelastic behavior of functional hydrogels formulated with liquors at representative autohydrolysis temperatures. It should be indicated that similar profiles and intermediate elastic (G′) and viscous (G″) moduli values were identified for the hydrogels made with the other liquors. For comparative purposes, the rheological profile of the corresponding potato starch hydrogel prepared at 10 g/L in distilled water is also displayed. All starch-based hydrogels featured characteristic gel behavior, exhibiting an elastic modulus higher than the viscous modulus and both almost frequency independent. At fixed frequency, two tendencies can be observed for the viscoelastic behavior of prepared hydrogels. For systems prepared with liquors from 50 to 170 °C, slight differences are observed in the magnitude of the viscoelastic modulus, showing a slight decreasing trend with increasing temperature. In the case of those prepared with liquors from 210 to 250 °C, G′ and G″ moduli notably dropped at fixed frequency with increasing autohydrolysis temperature. In all cases, lower values than those observed for the starch-based hydrogel in the absence of bioactive compounds was identified. Overall, it was clearly seen that the lowest viscoelastic characteristics were found for the gelling matrices formulated with the autohydrolysis liquors exhibiting the highest bioactive profiles. These outcomes are consistent with the softening of other biopolymer-based matrices incorporated with different bioactive compounds [28]. It should be highlighted that the softening observed in the presence of tested liquors was smaller than that reported for later authors, which can be related to the glucans present in the liquors that can favor the gelling behavior [21]. Nevertheless, the rheological profiles as well as the magnitudes for both elastic and viscous moduli identified hydrogels with an intermediate strength nature.

## 4. Conclusions

Autohydrolysis proves to be a suitable technology for the solubilization of valuable components for the food and nutraceutical market. A significant beneficial influence of temperature was observed. Maximum sugar yields were attained at 210 °C. The phenolic content and the antioxidant properties showed a continuous increase in the temperature range studied (up to 250 °C) with maximum yields of 3.4 g GAE/100 g dry raw material and 10.0 g Trolox eq/100 g dry raw material. Autohydrolysis is an efficient process for environmentally friendly processing for the selective solubilization of phenolic compounds with antiradical properties from *Lentinus edodes*. Furthermore, the viability of developing functional hydrogels with attractive rheological features offers novel potential possibilities for the valorization of the *Lentinus edodes* extracts.

## Figures and Tables

**Table 1:** Composition of *Lentinula edodes* in mass percentage over dry matter.

Compound	% over Dry Matter
Carbohydrates	54.1 ^a^
Glucan	28.7
Xylan	0.47
Galactan	1.46
Mannan	1.30
Fucan	0.68
Polyols	13.9
Uronic acids	4.48
Acetyl groups	3.22
Proteins	24.3 ^b^
Fatty acids	2.48 ^e^
Linoleic acid	1.93
Palmitic acid	0.55
Acid-insoluble matter	1.44 ^f^
Ashes	8.13 ^d^
Other	9.50 ^c^

Standard deviations were <2% in all cases. Data values in a column with different superscript letters are significantly different at the *p* ≤ 0.05 level.

**Table 2:** Reaction ordinate (R_o_) and severity for each experiment, calculated between the initial and final temperatures of 30 °C.

Exp	T max, °C	Mode	R_o_	Severity
1	50	Isotherm 7.5 min	0.5	−0.30 ^h^
2	80	Non-isotherm	1.5	0.18 ^g^
3	100	Non-isotherm	4.6	0.66 ^f^
4	130	Non-isotherm	26.5	1.42 ^e^
5	180	Non-isotherm	313.7	2.50 ^d^
6	210	Non-isotherm	3969	3.60 ^c^
7	230	Non-isotherm	17,610	4.25 ^b^
8	250	Non-isotherm	78,393	4.89 ^a^

Standard deviations were <5% in all cases. Data values in a column with different superscript letters are significantly different at the *p* ≤ 0.05 level.
